# Prophylactic Cranial Irradiation Following Surgical Resection of Early-Stage Small-Cell Lung Cancer: A Review of the Literature

**DOI:** 10.3389/fonc.2017.00228

**Published:** 2017-09-29

**Authors:** Brooke C. Bloom, Alexander Augustyn, Boris Sepesi, Sunil Patel, Shalin J. Shah, Ritsuko U. Komaki, Steven E. Schild, Stephen G. Chun

**Affiliations:** ^1^Trinity University, San Antonio, TX, United States; ^2^Division of Radiation Oncology, Department of Radiation Oncology, The University of Texas MD Anderson Cancer Center, Houston, TX, United States; ^3^Department of Thoracic and Cardiovascular Surgery, The University of Texas MD Anderson Cancer Center, Houston, TX, United States; ^4^Department of General Oncology, Division of Cancer Medicine, The University of Texas MD Anderson Cancer Center, Houston, TX, United States; ^5^Department of Radiation Oncology, Mayo Clinic Scottsdale, Scottsdale, AZ, United States

**Keywords:** small-cell lung cancer, early stage, surgical resection, prophylactic cranial irradiation, brain metastasis

## Abstract

With increasing use of low-dose screening CT scans, the diagnosis of early-stage small-cell lung cancer (SCLC) without evidence of mediastinal nodal or distant metastasis is likely to become more common, but the role of adjuvant therapies such as prophylactic cranial irradiation (PCI) are not well understood in this population. We performed a review of the literature pertaining to the impact of PCI in patients who underwent surgical resection of early-stage SCLC. Four studies were identified that were pertinent including three single-institution retrospective analyses and a National Cancer Database analysis. Based upon these studies, we estimate the rate of brain metastases to be 10–15% for Stage I and 15–25% for Stage II disease without PCI. However, the impact of PCI on the development of brain metastases and its ultimate impact on overall survival were not consistent across these studies. In summary, there is sparse evidence to guide recommendations for PCI following resection of early-stage SCLC. While it may be reasonable to offer PCI to maximize likelihood of cure, alternative strategies such as observation with close imaging follow-up can also be considered for the appropriate patient given the known neurocognitive side effects of PCI.

## Background

Small-cell lung cancer (SCLC) is a common smoking-related malignancy that accounts for approximately 15% of all lung cancers (1, 2). For limited-stage SCLC (3), combined modality therapy with concurrent chemotherapy and early thoracic radiation (TRT), followed by prophylactic cranial irradiation (PCI) is considered to be the standard of care (4–7). Although not commonly performed, surgical resection for selected patients with early-stage tumors without evidence of mediastinal nodal metastases may be reasonable. However, the role of adjuvant therapies such as PCI for surgically resected early-stage SCLC has not been formally studied in a prospective clinical trial.

For limited-stage SCLC treated with curative-intent definitive chemoradiation, multiple studies have demonstrated that PCI reduces brain metastases and improves overall survival dating back to the 1970s (2, 4, 8). The brain has long been established to be a sanctuary site for SCLC where there is poor chemotherapy penetration and roughly 50% of patients develop brain metastases (2, 9). However, in these studies, most patients had bulky and unresectable disease treated with chemoradiotherapy, and the applicability of this data to surgical resected early-stage SCLC is questionable. Moreover, the absolute survival benefit (5.4%) seen in the meta-analysis by Auperin et al. was small (4), suggesting that the benefit of PCI for surgically resected early-stage SCLC might be even smaller.

Historically, there have been few opportunities to study PCI for surgically resected SCLC. From a clinical standpoint, most limited-stage cases are not amenable for oncologic resection due to locally-advanced presentation. Furthermore, two historic trials did not demonstrate a clear role for surgery for SCLC (10, 11). For these reasons, there is little information available on surgical resection for SCLC and even less information available on the role of adjuvant therapy. For patients with early-stage SCLC (AJCC Stages I and II) who have undergone oncologic resection, the impact of adjuvant therapy such as PCI is debatable (12, 13). However, with increasing use of low-dose screening CT scans (LDCT), it is conceivable that patients with resected early-stage SCLC will become more common, particularly in regions that have high rates of tobacco use.

In this mini-review, we present the studies in the literature comparing outcomes of patients with and without PCI after surgical resection for early-stage (Stages I and II) SCLC. This included single-institution retrospective and a National Cancer Database (NCDB) analyses. The purpose of this review is to provide a concise resource to personalize recommendations for patients who have undergone surgical resection for early-stage SCLC.

## Methods

We performed a PubMed search using terms, “surgical resection,” “small-cell lung cancer,” “early-stage,” and “prophylactic cranial irradiation” to identify studies addressing the role of PCI for surgically resected SCLC. For the purpose of this mini-review, we excluded studies that did not include PCI. Using these criteria, three single institutional retrospective analyses (2, 14, 15), and a population-based analysis of the United States NCDB (16) were identified that compared outcomes of patients treated with and without PCI for surgically resected early-stage SCLC.

## Results

In reviewing the literature, three small single institution retrospective analyses were identified from the Tumor Hospital, Shan Dong Province, China (14), the Shanghai Chest Hospital, China (2), and the University of Heidelberg (15). In these studies, patients who underwent surgical resection for Stages I and III disease were compared with respect to whether they received PCI. A retrospective analysis of the United States NCDB that addressed PCI in this population with respect to overall survival was also identified (16). Rates of brain metastases reported in these studies are summarized in Table 1.

**Table 1:** Brain metastasis rates reported in the literature for surgically resected early-stage small-cell lung cancer.

Citation	Brain metastasis rate
Xu *et al*. (2), Shanghai Chest Hospital	Stage I—13.6% (no PCI) vs. 10.5% (PCI)
	Stage II—22.4% (no PCI) vs. 12.8% (PCI)
Zhu et al. (14), Shandong Cancer Hospital	Stage I (no PCI)—9.4%
	Stage II (no PCI)—18.2%
Bischof et al. (15), University of Heidelberg	Stages I and II combined—22% (no PCI) vs. 0% (PCI)

*PCI, prophylactic cranial irradiation*.

In the largest study by Xu et al. (2) from the Shanghai Chest Hospital, 349 patients were analyzed, of whom 115 received PCI and 234 did not receive PCI. Approximately half (*N* = 189) of the patients had Stages I and II disease for whom the association of PCI on oncologic outcomes is summarized in Table 1. For Stage I SCLC, patients who received PCI had no survival advantage (HR 1.61, 95% CI 0.68–3.83) or associated reduction in development of brain metastases (13.6 vs. 10.5%). For Stage II SCLC, PCI was associated with an overall survival benefit on multivariable analysis (HR 0.54, *p* = 0.047), as well as a statistical trend toward reduction in brain metastases (22.4 vs. 12.8%, *p* = 0.094).

In another single institution analysis from the Tumor Hospital, Shan Dong, China, 193 patients were analyzed with respect to delivery of PCI after surgical resection for Stages I–III SCLC (14). While PCI was associated with a survival and brain metastasis free survival benefit in all patients, subgroup analysis of Stage I patients showed no survival benefit associated with PCI. PCI was associated with a twofold reduction in brain metastases with 9% of developing them in the PCI group and 22% in the non-PCI group. The non-PCI brain metastasis rates were listed as 9.4% for Stage I and 18.2% for Stage II. Further subgroup analysis comparing brain metastases rates by stage groups was not reported.

The smallest study evaluating the impact of PCI on rates of brain metastases from the University of Heidelberg reported 39 patients who underwent resection for Stages I and II SCLC from 1995 to 2006 (15). This study contains the additional confounding factor in that it sought to evaluate both the role of adjuvant thoracic radiation therapy (TRT) as well as the role of PCI on a very small number of patients. PCI was administered to a total dose of 28–30 Gy in standard fractions of 2 Gy daily. In this study, 44% of patients received no form of radiation, while 15% received PCI alone, 3% received TRT alone, and 38% received both PCI and TRT. Rates brain metastases were grouped for all Stages I and II patients with a 22% brain metastasis rate for patients without PCI and no brain failures reported in the PCI group. The authors reported that PCI had a significant (*p* = 0.01) survival benefit although the magnitude of this benefit is not reported.

From the United States, an NCDB analysis was performed on patients treated with surgical resection for T1-T2N0 SCLC from 2003 to 2011 (16). In this study, 99 patients (52.1%) of patients received radiation therapy to the brain, which was interpreted as PCI delivery. On multivariable analysis, radiation was not associated with a significant survival benefit when used either alone or in conjunction with chemotherapy. Information on rates of brain metastases was not reported because such information is not captured in the NCDB.

## Discussion

Currently, the best available information on PCI for early-stage SCLC is based upon underpowered retrospective analyses that do not set a clear precedent for standard of care. These retrospective studies help us estimate the brain metastasis rate for early-stage SCLC to be roughly 10–15% for Stage I disease and for 15–25% for Stage II disease. PCI is known to cause neurocognitive side effects (17), and the overall survival benefit is likely to be less than 5% based upon extrapolation from unresectable SCLC (4). Without a clear standard of care regarding PCI in these patients, we advocate multidisciplinary evaluation and patient-tailored recommendations. While offering PCI may be reasonable to maximize likelihood of cure, close interval follow-up using serial brain MRIs may also be a reasonable strategy in the compliant patient.

Exploration of PCI for resected early-stage SCLC may represent an opportunity for investigation with the implementation of LDCT. Multiple prospective trials lead the conclusion from the United States Preventative Task Force that LDCT reduce lung cancer mortality (18). With increasing use of screening, thoracic surgeons using video-assisted thorascopic surgery for diagnostic wedge resection may also increase diagnoses of early-stage SCLC. Analyses of the Surveillance, Epidemiology, and End Results Program database have also supported a role for surgical resection for this population (19, 20). As such, especially in geographic regions where tobacco use remains prevalent, early-stage resected SCLC may become more common with increased screening and early detection. While a formal randomized trial for this situation may not be feasible, it may be possible to develop a prospective registry to better understand oncologic outcomes in these patients. Data from such a registry might also provide useful information in conjunction with molecular profiling to shed light on the need for adjuvant treatments such as PCI, immune therapy, or cytotoxic chemotherapy.

Although PCI has been established to cause neurocognitive side effects in a substantial number of patients, emerging strategies may mitigate the risk of this toxicity. The *N*-methyl-d-aspartate inhibitor memantine has previously shown to improve neurocognitive side effects from whole-brain radiation therapy for patients with overt metastases (21). Intensity-modulated radiation therapy has also been employed with hippocampal avoidance specifically for the purpose of improving neurocognition (22, 23). These strategies have been combined in NRG Oncology CC-001 with the intent of improving quality of life and cognition for patients receiving prophylactic whole-brain radiation. Results of NRG Oncology CC-001 will be helpful in determining whether PCI with hippocampal avoidance is a reasonable strategy to prevent brain failures while minimizing neurocognitive consequences.

In summary, there is little information currently available on PCI for resected early-stage SCLC. With increased screening, these patients may represent a new frontier for investigation. It may be reasonable to offer PCI for the purpose of minimizing intracranial recurrence rate while counseling patients that the reduction in brain metastases and improvement in survival is likely to be small. While the side effects of whole-brain PCI may be unpalatable to some patients, strategies such as memantine and hippocampal avoidance have potential to mitigate the toxicities of PCI and should be explored enthusiastically.

## Author Contributions

BB and AA: contributed to data collection and writing of manuscript. SJS, BS, and RK: contributed to data interpretation and writing of manuscript. SES and SC: contributed to data interpretation, data collection, and writing of manuscript.

## Conflict of Interest Statement

The authors declare that the research was conducted in the absence of any commercial or financial relationships that could be construed as a potential conflict of interest.
